# The Association Between FGF21 and Diabetic Erectile Dysfunction: Evidence from Clinical and Animal Studies

**DOI:** 10.3389/fendo.2022.874796

**Published:** 2022-06-15

**Authors:** Song Yang, Yichun Zhang, Xiaohui Lyu, Yuanyuan Gu, Guodong Zhang, Pengcheng Liu, Yulu Zheng, Zheng Guo, Yanbo Zhang, Haifeng Hou

**Affiliations:** ^1^ Department of Endocrinology, Taian City Central Hospital, Taian, China; ^2^ School of Public Health, Shandong First Medical University and Shandong Academy of Medical Sciences, Taian, China; ^3^ Department of Outpatient Department, Taian City Central Hospital, Taian, China; ^4^ Department of Pharmacy, Taian City Central Hospital, Taian, China; ^5^ Department of Endocrinology, Feicheng Hospital Affiliated to Shandong First Medical University, Taian, China; ^6^ Centre for Precision Health, School of Medical and Health Sciences, Edith Cowan University, Perth, WA, Australia; ^7^ The Second Affiliated Hospital of Shandong First Medical University, Taian, China

**Keywords:** fibroblast growth factor 21, erectile dysfunction, diabetes mellitus, diagnosis, clinical study, rat

## Abstract

Erectile dysfunction (ED), a complication of diabetes mellitus (DM), affects 50–75% of men with diabetes. Fibroblast growth factor 21 (FGF21) is a liver-derived metabolic regulator which plays a role in insulin-independent glucose uptake in adipocytes. We designed a clinical study and an animal experiment to investigate the relationship between FGF21 and DM-induced ED. The clinical study enrolled 93 participants aged > 18 years (61 patients with type 2 DM and 32 healthy controls) from Taian City Central Hospital (TCCH) in Shandong Province, China, amongst whom the association between serum FGF21 and diabetic ED was analyzed. To further validate this association, we developed animal model of diabetic ED using Sprague-Dawley (SD) rats. Serum FGF21 concentration and FGF21 mRNA expression in penile samples of the rats were determined with Western blotting and quantitative real-time PCR. Among the 93 participants, the level of serum FGF21 was negatively correlated with the IIEF-5 score (*r* = -0.74, *P* < 0.001). The analysis on the performance of FGF21 for ED diagnosis showed that the area under the receiver operating characteristic (ROC) curve was 0.875 (95% confidence interval [CI]: 0.803 to 0.946). In the animal experiment, the levels of serum FGF21, 2^-Δ Δ Ct^ values of FGF21 mRNA expression, and relative levels of FGF21 in penile samples were higher in the ED group compared to the DM and control groups. Our findings demonstrated an association between the FGF21 level and diabetic ED, indicating the potential of this cytokine in predicting diabetic ED.

## Introduction

The prevalence of diabetes mellitus (DM) has been growing at an alarming speed, affecting 8.4% of adults in 2017, accounting for 9.9% of global all-cause death (1). As a metabolic disorder resulting from defective insulin secretion and insulin action, DM is characterized by a state of hyperglycemia (2). The complications of DM, including vascular disease, neuropathy, nephropathy, eye disease, and erectile dysfunction (ED), are the major sources of DM-related mortality and morbidity (3, 4). DM-induced erectile dysfunction (DMED) impacts roughly 50–75% of men with DM (5, 6). The prevalence of ED is three times higher in diabetic men versus the general male population, among whom the onset of ED is up to 10 years earlier than those without diabetes (7). The development of ED is a multifactorial process involving changes in vascular dilatation capacity, peripheral sympathetic activity, and endothelial function (5). Hyperglycemia is one of the contributors of endothelial lesions and vasculogenic dysfunction, which are the main causes of DMED (2).

The fibroblast growth factor (FGF) family consists of 22 members that display multiple biological functions, such as improving insulin sensitivity, glucose and lipid metabolism (8, 9). FGF21, mainly expressed in the liver and adipocytes, plays a role in ameliorating glucose tolerance and insulin sensitivity *via* regulating glucose uptake in adipocytes and restraining glucose production in hepatocytes (10, 11). Research has demonstrated that FGF21 protects a variety of cells (*e.g.*, islet β-cells, endothelial cells, cardiomyocytes, and dopaminergic neurons) from acute injuries (12). In addition, studies have reported the preventive effect of FGF21 on atherosclerosis and coronary artery disease (CAD) through protecting endothelial function and antioxidation (13, 14). Since endothelial dysfunction is one of the pathophysiological pathways of ED, it is hypothesized that FGF21 is associated with ED in men with diabetes. Here we conducted a clinical study and an animal experiment to investigate the association between FGF21 and diabetic ED.

## Methods

### Study Participants

We randomly recruited 31 adult men with type 2 diabetes mellitus (T2DM), 30 patients with DMED and 32 healthy controls from Taian City Central Hospital (TCCH) in Shandong Province between January 2017 and June 2018.

Inclusion criteria of T2DM patients were as follows: (1) diagnosed in accordance with American Diabetes Association criteria (15); (2) age > 18 years; (3) not enrolled in other clinical studies; (4) the International Index of Erectile Function (IIEF-5) score > 21. Inclusion criteria of DMED patients were as follows: (1) diagnosed in accordance with American Diabetes Association criteria; (2) age > 18 years; (3) not enrolled in other clinical studies; (4) IIEF-5 score≤21. Exclusion criteria were as follows: (1) patients with severe diseases, such as cardiovascular disease (CVD), stroke, cancers, hypothyroidism, tuberculosis, or communicable diseases; and (2) patients with psychiatric conditions. The Ethical Committee of TCCH approved this study (No. 2017-06-27). Informed consent was obtained from each study participant.

### Physical and Biochemical Examinations

Participants’ demographics, disease history, and anthropometric data were collected through face-to-face interviews. Trained clinicians examined participants’ external genitalia (i.e., penis shape, size, nerve reflexes, and skin sensations). IIEF-5 was developed to identify the presence or absence of ED on the basis of the National Institute of Health (NIH) definition of ED. The five items reflected erectile function and intercourse satisfaction of respondents (16). The degree of ED was defined based on the IIEF-5 score: severe ED (0–7), moderate ED (8–11), mild ED (12–21), and normal function (22–25) (17, 18).

All participants were asked to fast overnight for ≥ 10 h before blood samples were taken. Serum FGF-21 levels were determined using an enzyme-linked immunosorbent assay (ELISA) kit (Kanglang Biotechnology, Shanghai, China). Fasting blood glucose (FBG) was measured with an automatic analyzer (Hitachi, Tokyo, Japan). Glycated hemoglobin A1c (HbA1c) was detected *via* high-performance liquid chromatography (Bio-Rad Laboratories, CA, USA). Total cholesterol (TC), triglycerides (TG), mean platelet volume (MPV), low-density lipoprotein cholesterol (LDL-C), and high-density lipoprotein cholesterol (HDL-C) levels were determined with an automatic analyzer (Hitachi, Tokyo, Japan). Fasting insulin (FINS) and testosterone were measured *via* electrochemiluminescence assay (Roche Diagnostics, Basel, Switzerland). Homeostasis model assessment of insulin resistance (HOMA-IR) was conducted based on the following formula:


HOMA-IR=FINS(mU/L)×fasting glucose (mmol/L)/22.5

### Animals

The association between FGF21 and diabetic ED was validated in specific pathogen-free (SPF) Sprague-Dawley (SD) rats. Forty male rats aged 8 weeks were provided by Hunan Slac Jingda Laboratory Animal Co., Ltd (Changsha, China). The 10 rats in the control group were treated with an injection of 0.9% normal saline (NS). To develop T2DM models, 30 rats were randomly selected and treated with streptozotocin at 60 mg/kg body weight *via* intraperitoneal injection. The rats that had a fasting blood glucose level ≥ 16.7 mmol/L on the 3^th^ and 7^th^ day after the injection were defined diabetic. Twelve weeks later, 25 of the 30 rats injected with streptozotocin had survived. Five rats that died during streptozotocin administration were excluded from this study. Then apomorphine (APO) test was conducted to assess erectile function in rats. After weighing, erectile responses were assessed by injection of APO (80 mg/kg, subcutaneously) in the loose skin of the back of the neck. Then the rats were placed in a dark and quiet room for 30 min, and the number of erections was recorded. A penile erection was regarded to occur complete emergence of an engorged glans penis and distal penile shaft. Twelve rats without penile erection were diagnosed as DMED rats (19).

Penile tissues were obtained from the rat post-executed by dislocation, and stored in 10% paraformaldehyde solution at -80 °C for further examinations. The FGF21 levels in serum in the DM, DMED, and control groups were detected with an ELISA kit (Cusabio Biotech CO. LTD, Wuhan, China) according to the manufacturer’s instructions.

### Western Blotting

The target protein in the rat penile samples was analyzed using Western blotting assay. Briefly, triturated frozen penile samples were lysed on ice for 30 min in lysis buffer (150 mM NaCl, 20 mM Tris-HCl, pH 7.4, 0.1% SDS, 1.0% NP-40, 0.5% Na-deoxycholate, 0.2 mM PMSF, and protease inhibitor cocktails). The lysates were centrifuged at 12,000 r for 20 min and separated in 10% polyacrylamide gel. The samples were next transferred to polyvinylidene fluoride (PVDF) membranes, blocked with 5% skimmed milk for 60 min, and probed with the primary antibodies of FGF21 at 4°C overnight. The blots were then incubated with horseradish peroxidase–conjugated anti-IgG for 1h at room temperature and detected using ECL Western Blotting Detection Reagents (Thermo Scientific Pierce, Rockford, IL, USA).

### Quantitative Real-Time PCR

Quantitative real-time PCR (qRT-PCR) was employed to measure relative levels of FGF21 mRNA. Briefly, total RNA was isolated from the penile samples with Trizol reagent (Invitrogen, Grand Island, NY, USA). The amount of RNA in each sample was identified by absorbance at 260 nm. The following primers synthesized by TaKaRa (Dalian, China) were used: forward primer (5’-GGGTCAAGTCGACAGGTAT-3’) and reverse primer (5’- ATCAAAGTGGAGGCGATAGA-3’). The cDNAs were synthesized with MuLV Reverse Transcriptase (Applied Biosystems, CA, USA). The qRT-PCR method was used with a Rotor-Gene Q real-time PCR cycler (Qiagen, Germany) and a SYBR-green PCR master mix kit (Applied Biosystems, CA, USA). Relative mRNA levels were quantified using the 2^−ΔΔCt^ method and normalized *via* β-Actin mRNA. Each experiment was repeated five times.

### Statistical Analysis

The Kolmogorov-Smirnov test was used to check the normal distribution of variables. Continuous data that were normally distributed were presented as means and standard deviations (SDs). The statistical significance between groups was tested using one-way analysis of variance (ANOVA), followed by the Student–Newman–Keuls (SNK) test and Pearson’s correlation. Continuous variables with a non-normal distribution were represented as the median (P25-P75) and were compared with the Wilcoxon rank-sum test. Spearman correlation analysis was used to calculate the correlation coefficient between IIEF-5 scores and relevant parameters for all participants. The multiple linear regression model and Logistics regression analyses were used to analyze the DMED-related factors. The receiver operating characteristic (ROC) curve was modeled to analyze the performance of circulating FGF21 levels for DMED diagnosis. Statistical analyses were carried out in SPSS software (v25.0, SPSS Inc. Chicago, USA). A probability value of less than 0.05 was considered statistically significant for each test.

## Results

### Characteristics of Study Participants

The clinical characteristics of all participants enrolled in the human study appear in **Table 1**. Among the 61 diabetic men, the 30 with ED were enrolled in the DMED group; the 31 participants without ED were in the DM group. Among the 30 participants in the DMED group, 7 ED cases (23.3%) were severe, 8 (26.7%) were moderate, and 15 (50%) were mild.

**Table 1:** Characteristics of study participants in human study.

Variables	DMED(n = 30)	DM(n = 31)	Control(n = 32)	*F/Z/χ^2^*	*P*
Age (years)	49.83 ± 6.14	48.32 ± 6.69	48.31 ± 4.94	0.658	0.521
Education years	11.57 ± 3.64	11.97 ± 3.75	11.06 ± 3.44	0.499	0.609
Smoking (%)	11 (36.7)	11 (35.5)	12 (37.5)	0.028	0.986
Alcohol drinking (%)	12 (40.0)	11 (35.5)	10 (31.3)	0.518	0.772
BMI (kg/m^2^)	25.15 ± 3.01	24.63 ± 3.69	24.34 ± 2.34	0.548	0.580
SBP (mmHg)	134.10 ± 9.07 ^#^	131.06 ± 8.85	128.06 ± 6.79	4.118	0.019
DBP (mmHg)	84.97 ± 7.39 ^#^	80.35 ± 8.50	79.81 ± 5.84	4.586	0.013
HbA1c (%)	8.09 ± 1.02 ^#^	7.96 ± 1.39 ^#^	5.30 ± 0.28	77.753	<0.001
FBG (mmol/l)	8.51 ± 1.15 ^#^	8.02 ± 1.02^#^	5.13 ± 0.37	126.196	<0.001
TC (mmol/L)	4.97 ± 0.53 ^#^	4.76 ± 0.52 ^#^	4.11 ± 0.30	29.751	<0.001
TG (mmol/L)	2.13 ± 0.39 ^#^	1.96 ± 0.32 ^#^	1.35 ± 0.29	47.346	<0.001
LDL-C (mmol/L)	2.97 ± 0.43 ^#^	2.82 ± 0.31 ^#^	2.24 ± 0.34	35.816	<0.001
HDL-C (mmol/L)	1.13 ± 0.23 ^#^	1.11 ± 0.25 ^#^	1.30 ± 0.25	6.153	0.003
FGF21(pg/mL)	112.46 ± 9.16^*#^	103.97 ± 6.20 ^#^	91.94 ± 3.89	73.372	<0.001
Testosterone (ng/ml)	5.02 ± 0.62 ^#^	5.25 ± 0.55 ^#^	6.25 ± 0.42	46.833	<0.001
FINS (uIU/ml)	9.06 ± 1.94 ^#^	8.58 ± 1.23 ^#^	7.88 ± 1.05	5.158	0.008
MPV (fL)	9.83 ± 1.51 ^#^	9.28 ± 1.18 ^#^	8.62 ± 0.75	8.200	0.001
HOMA-IR	3.44 ± 0.91 ^#^	3.07 ± 0.63 ^#^	1.8 ± 0.25	54.765	<0.001
IIEF-5 scores	12.00(7.75,16.00)^*#^	23.00(22.00,23.00)^*#^	24.00(23.00,24.00)	70.385	<0.001

BMI, body mass index; DBP, Diastolic blood pressure; FBG, Fasting blood glucose; FGF21, Fibroblast growth factor 21; FINS, Fasting insulin; HbA1c, Hemoglobin A1c; HDL-C, high-density lipoprotein cholesterol; HOMA-IR, homeostasis model assessment of insulin resistance; IIEF-5, international index of erectile function-5; LDL-C, low-density lipoprotein cholesterol; MPV, mean platelet volume; TC, cholesterol; TG, triglycerides; SBP, Systolic blood pressure; ^*^P < 0.05 compared with DM group; ^#^P < 0.05 compared with the controls.

No significant differences were identified in participants’ age, alcohol consumption, smoking habits, education level, and body mass index between the DMED, DM, and healthy control groups. Levels of serum FGF21 in the DMED group were significantly higher than in the DM and control groups (*P* < 0.05). Levels of HbA1c, systolic blood pressure (SBP), diastolic blood pressure (DBP), FBG, TC, TG, HDL-C, LDL-C, HOMA-IR, MPV and FINS were higher in the DMED group than in the control group (*P* < 0.05). No differences were identified between the DMED and DM groups. Testosterone and IIEF-5 score were significantly lower in DMED cases compared to controls (*P* < 0.05).

### Correlation Between Serum FGF21 Level and IIEF-5 Score

**Figure 1:**
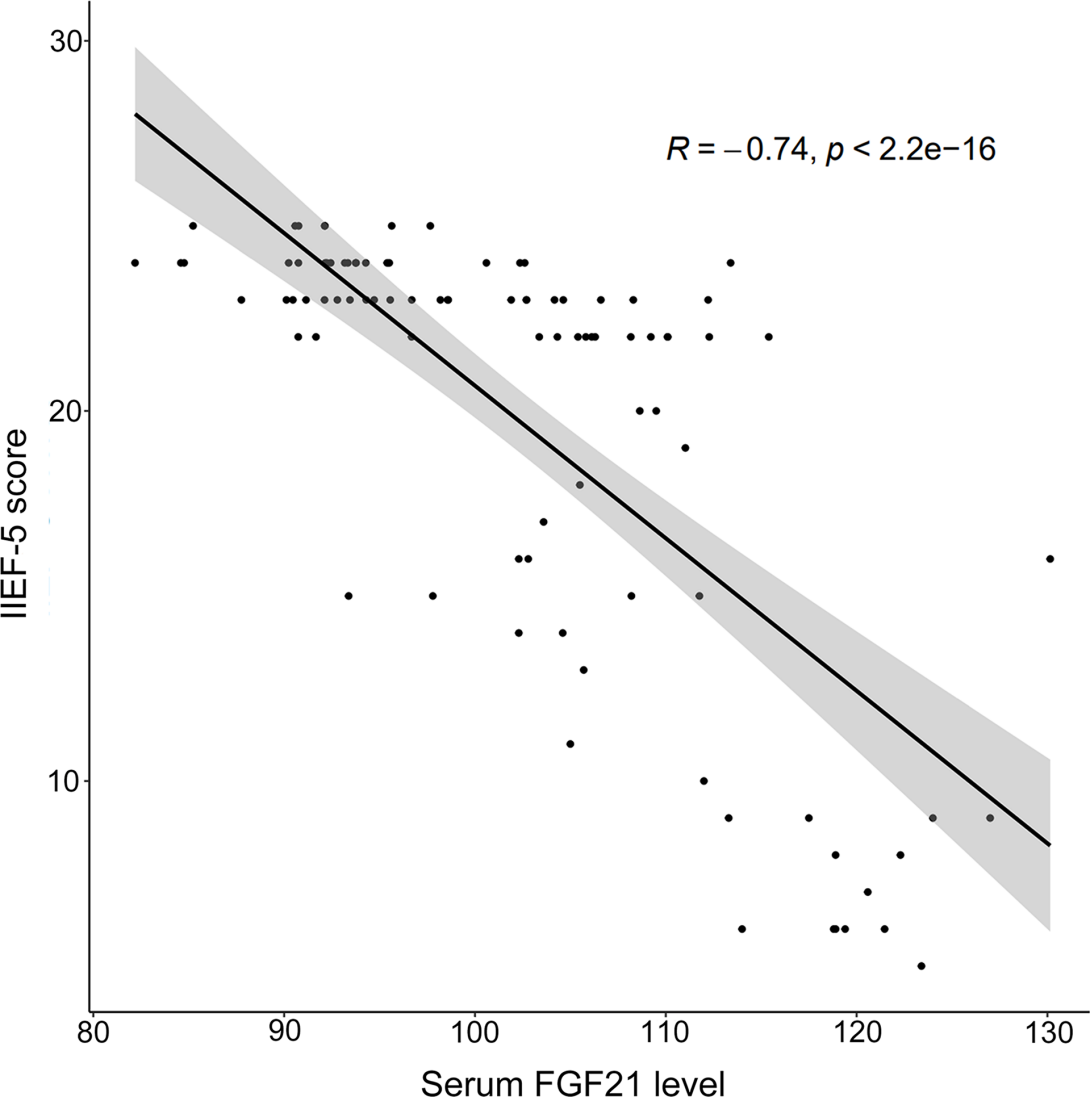
The Spearman correlation between serum FGF21 and IIEF-5 score in human study.

A spearman correlation analysis was conducted to explore the relationships among IIEF-5 scores and relevant parameters for all participants. As depicted in **Figure 1**, a negative correlation was observed between the serum FGF21 level and IIEF-5 score (*r_s_* = -0.738, *P* < 0.001). As listed in **Table 2**, SBP, DBP, HbA1c, FBG, TC, TG, HOMA-IR, FINS, MPV, and LDL-C were negatively correlated with IIEF-5 scores. Moreover, the serum FGF21 level was negatively correlated with HDL (*r* = -0.414, *P* < 0.001) and testosterone (*r* = -0.716, *P <* 0.001), respectively. Meanwhile, the serum FGF21 level was positively correlated with SBP (*r* = 0.386, *P* < 0.001), DBP (*r* = 0.336, *P =* 0.001), HbA1c (*r* = 0.656, *P <* 0.001), FBG (*r* = 0.749, *P <* 0.001), TC (*r* = 0.676, *P <* 0.001), TG (*r* = 0.712, *P <* 0.001), LDL-C (*r* = 0.694, *P <* 0.001), MPV (*r* = 0.374, *P <* 0.001), HOMA-IR (*r* = 0.675, *P <* 0.001), and FINS (*r* = 0.293, *P =* 0.004), respectively.

**Table 2:** Correlation analysis on serum FGF21 level and IIEF-5 score in human study.

Variables	IIEF-5		FGF21	
	*r_s_*	*P*	*r*	*P*
Age (year)	-0.108	0.303	0.062	0.552
Education year	-0.104	0.323	0.052	0.619
Smoking	0.034	0.745	-0.005	0.963
Alcohol drinking	0.070	0.505	0.063	0.550
BMI (kg/m^2^)	-0.236	0.023	0.123	0.240
SBP (mmHg)	-0.388	<0.001	0.386	<0.001
DBP (mmHg)	-0.320	<0.001	0.336	0.001
HbA1c (%)	-0.698	<0.001	0.656	<0.001
FBG (mmol/l)	-0.716	<0.001	0.749	<0.001
TC (mmol/L)	-0.641	<0.001	0.676	<0.001
TG (mmol/L)	-0.688	<0.001	0.712	<0.001
LDL-C (mmol/L)	-0.645	<0.001	0.694	<0.001
HDL-C (mmol/L)	0.299	0.004	-0.414	<0.001
Testosterone (ng/ml)	0.706	<0.001	-0.716	<0.001
FINS (uIU/ml)	-0.397	<0.001	0.293	0.004
MPV (fL)	-0.305	0.003	0.374	<0.001
HOMA-IR	-0.665	<0.001	0.675	<0.001

BMI, body mass index; DBP, Diastolic blood pressure; FBG, Fasting blood glucose; FGF21, Fibroblast growth factor 21; FINS, Fasting insulin; HbA1c, Hemoglobin A1c; HDL-C, high-density lipoprotein cholesterol; HOMA-IR, homeostasis model assessment of insulin resistance; IIEF-5, international index of erectile function; LDL-C, low-density lipoprotein cholesterol; MPV, mean platelet volume TC, cholesterol; TG, triglycerides; SBP, Systolic blood pressure.

### Multiple Linear Regression and Logistics Regression Analyses on DMED-Related Factors

A multiple linear regression model was established to screen DMED-related factors. As shown in **Table 3**, FGF21 was one of the independent determinants significantly associated with the IIEF-5 score.

**Table 3:** Multivariate linear regression analysis on the influencing factors of IIEF-5 score in human study.

Variables	*B*	*SE*	*β*	*t*	*P*
FGF21	-0.229	0.061	-0.410	-1.770	0.000
HOMA-IR	-1.161	0.656	-0.185	-0.466	0.080
Testosterone	1.284	0.896	0.161	-3.735	0.156
TC	-0.545	1.170	-0.053	3.996	0.643
DBP	-0.120	0.077	-0.152	-0.005	0.122
SBP	0.000	0.073	-0.001	1.433	0.996

DBP, Diastolic blood pressure; FGF21, Fibroblast growth factor 21; HOMA-IR, homeostasis model assessment of insulin resistance; IIEF-5, international index of erectile function; TC, cholesterol; SBP, Systolic blood pressure; SE, standard error.

In order to evaluate the effects of these factors on DMED, we performed multiple logistic regression analyses. As shown in **Table 4**, the odds ratios (ORs) and 95% confidence intervals (CIs) were 1.193 (1.073 to 1.326) for FGF21, and 10.765 (2.277 to 50.895) for HOMA-IR and 24.373 (1.007to 610.416) for HDL.

**Table 4:** Logistic regression analysis on the determinants of erectile function in human study.

Variables	*B*	*SE*	*Walds*	*P*	*OR*	95% CI of OR	
						LCI	UCI
FGF21	0.177	0.0540	10.699	0.001	1.193	1.073	1.326
HOMA-IR	2.376	0.7926	8.988	0.003	10.765	2.277	50.895
HDL	3.211	1.6345	3.858	0.049	24.373	1.007	610.416

B, regression coefficient; CI, confidence interval; FGF21, Fibroblast growth factor 21; HDL, high-density lipoprotein; HOMA-IR, homeostasis model assessment of insulin resistance; LCI, lower confidence interval; OR, odds ratio; SE, standard error. SE, standard error; UCI, upper confidence interval.

### Role of FGF21 in DMED Diagnosis

**Figure 2:**
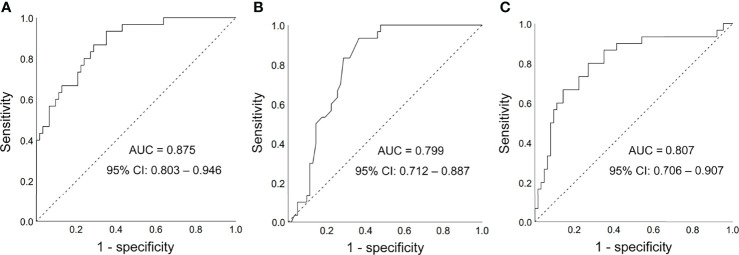
Receiver operating characteristic (ROC) curves. **(A)** FGF21 for diagnosis of DMED; **(B)** HbA1c for diagnosis of DMED; **(C)** HOMA-IR for diagnosis of DMED; AUC, area under the curve; CI, confidence interval.

To detect the performance of FGF21 for discrimination of DMED from DM patients and control group, we established a ROC curve. As presented in **Figure 2A**, the area under the curve (AUC) of FGF21 was 0.875 (95% CI: 0.803 to 0.946 P *<* 0.001). A cut-off value of FGF21 (102.75 pg/ml) was selected with a sensitivity of 0.867 and a specificity of 0.714. The AUC value was 0.799 (95% CI: 0.712 to 0.887, *P* < 0.001) in the model established with HbA1c, while it was 0.807 (95% CI: 0.706 to 0.907, *P* < 0.001) in the model established with HOMA-IR an (**Figures 2B, C**). We also established a model with testosterone, and the AUC was 0.772 (95% CI: 0.675 to 0.870, *P* < 0.001) (**Figure S1**).

### Serum FGF21 Level in DMED Rats

**Figure 3:**
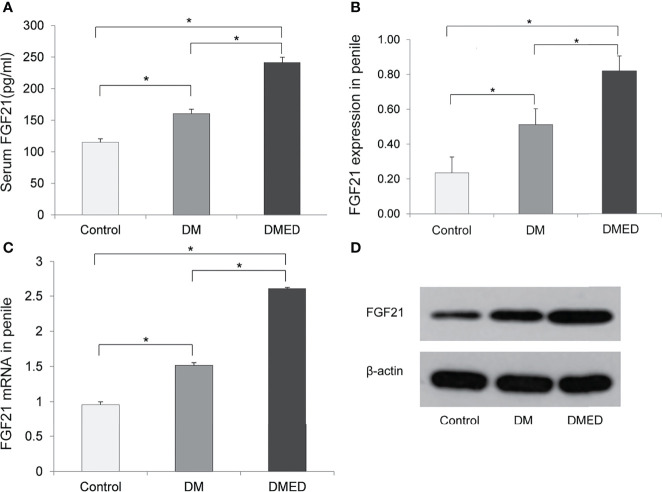
The expression of FGF21 and FGF21 mRNA in rat model. **(A)** The level of serum FGF21; **(B)** The relative level of FGF21 protein in penile samples; **(C)** The relative level of FGF21 mRNA in penile samples; **(D)** The representative result of western blotting assay of FGF21 in penile samples. The data shown in the graphs represent the mean ± SD, ^*^ *P* < 0.05.

**Figure 3A** indicates that FGF21 levels were significantly higher in the DMED group (241.12 ± 8.5 pg/ml) than in the DM (160.32 ± 7.2 pg/ml) and control (114.91 ± 5.6 pg/ml) groups. This finding was consistent with our human study.

### Expression of FGF21 Protein in Rat Penis

Western blotting assays revealed that the mean level of FGF21 in the penis was higher in DMED rats (0.82 ± 0.08) versus rats in the DM (0.51 ± 0.09) and control (0.23 ± 0.09) groups (**Figures 3B, D**).

### Level of FGF21 mRNA in Rat Penis

As shown in **Figure 3C**, the relative level of FGF21 mRNA as measured by 2^-△△CT^ values in the penis was significantly higher in the DMED group (2.61 ± 0.04) than in DM rats without ED (1.51 ± 0.04) and the controls (0.95 ± 0.02).

## Discussion

We investigated the relationship between FGF21 and diabetic ED in populations with T2DM and in diabetic rat models. Results of the human study demonstrated that the serum FGF21 level correlated with the IIEF-5 score. The International Index for Erectile Function (IIEF) is a patient-reported outcome measure, widely used in urology to measure erectile dysfunction (ED), applied both in clinical research and in daily clinical practice (16, 18, 20). Higher levels of FGF21 were observed in both humans and in rats with DMED compared to those with normal erectile function. Moreover, rats with DMED had increased expression of FGF21 protein and FGF21 mRNA compared to rats with normal erectile function, which validated our findings in humans.

ED is a complication that significantly harms the quality of life and psychological well-being of men with diabetes (21). Compared to DM treated with insulin, ED usually worsens in individuals with poor blood glucose control (2). Hyperglycemia is a major contributor to endothelial dysfunction, namely by disrupting vascular endothelial cells’ homeostasis and altering eNO-dependent vasodilation processes (22). Under physiologic conditions, insulin promotes relaxation of the precapillary sphincter which induces vasodilation by directly stimulating the expression and activation of NO synthase (23, 24). An aberrant vascular response to insulin and subsequent vasoconstriction have been observed in individuals with insulin resistance; these issues may contribute to ED (23, 25). A mendelian randomization study showed that T2DM has a causal relationship with ED independently from obesity and dyslipidemia (26).

Our study also showed that the serum FGF21 level is correlated with HOMA-IR and fasting glucose. This finding is consistent with prior studies (27–29). The administration of recombinant FGF21 in animal models has been deemed effective in ameliorating insulin sensitivity and reducing serum glucose levels (30, 31). FGF21 exerts direct effects on increased glucose uptake in skeletal muscle and insulin-stimulated glucose transport, which may contribute to glucose homeostasis (32). In addition, FGF21 promotes insulin secretion in islet β cells through the PI3K/Akt signaling pathway (33). The high levels of serum FGF21 observed in diabetic patients may be a compensatory response to decreased insulin sensitivity or potential FGF21 resistance (29, 34). FGF21 levels have been shown to be elevated 2–3 fold in oral glucose tolerance tests for patients with metabolic syndrome but not among healthy subjects (35).

Apart from the function of modulating energy metabolism, FGF21 plays a significance effect on vascular endothelium (36, 37), and is understood as a biomarker for subclinical vascular lesions (38). In addition, FGF21 stimulates the expression of adiponectin which suppresses proliferation and migration of smooth muscle cells and reduces uptake of oxidized LDL (39). Thus, FGF21 alleviates cardiovascular risk factors *via* improved lipid profiles. FGF21-induced activation of the CaMKK2-AMPKα signaling pathway suppresses oxidative stress and enhances endothelium-dependent vasorelaxation of the aorta, thus alleviating endothelial dysfunction (40). FGF21 directly prevents endothelial progenitor cell (EPC) damage induced by high glucose and promotes ischemic angiogenesis by increasing NAD+ content in an AMPK-dependent manner (10). FGF21 can also enhance EPC mobilization and angiogenic function under diabetic conditions (41). Vasculogenic is one type of ED including arteriogenic ED, venogenic ED and mixed vasculogenic ED (42). Studies have investigated the role of mean platelet volume (MPV) in diagnosis of vasculogenic ED, and patients with vasculogenic ED showed higher MPV compared with non-vasculogenic ED patients (43–45). Our findings showed that MPV level is not higher in the DMED group compared to the diabetic men without ED. It should be noted that in the clinical studies investigating the level of MPV among vasculogenic ED, the patients with diabetes were excluded (42, 46). However, in our study the ED patients were with diabetes.

Studies have also documented that FGF21 inhibits endothelial cell apoptosis and promotes DNA synthesis and differentiation of endothelial cells, which has been verified in the development of atherosclerosis (47, 48). Endothelial cells play a crucial role in maintaining erectile function by regulating contractions in the arteries of the corpus cavernosum and smooth muscle (49). The penile endothelium is a specialized extension of the vascular system (50). An impaired vascular endothelium induced by oxidative stress can cause ischemia and hypoxia of the corpus cavernosum. These conditions lead to cavernous smooth muscle damage and fibrosis, which result in DMED (51). Abnormal increase in FGF21 levels has been consider as a signal of endothelial cell injury (52) as reported in the diseases including obesity, dyslipidemia, metabolic syndrome, hypertension, CAD, DM, and nonalcoholic fatty liver (53–55). FGF21 can reduce oxidative damage and protects against high glucose–induced declines in cell viability (e.g., endothelial cell) (41). In addition, FGF21 is found to play a role in protection against eNOS dysfunction in endothelial cells exposed to high glucose (27).

## Limitations

This study did not investigate the specific pathophysiological mechanism between diabetic ED and FGF21. The possible beneficial effect of FGF21 in DMED treatment was not yet explored. Further studies are needed to clarify the potential metabolism and explore the therapeutic effect of FGF21 on DMED.

## Conclusions

Our study evidenced that FGF21 is closely associated with diabetic ED. Even though the pathophysiological mechanism has not been fully explored, our findings display the potential role of FGF21 in the development of diabetic ED. FGF21 could be employed as an indicator of ED in diabetic men.

## Data Availability Statement

The raw data supporting the conclusions of this article will be made available by the authors, without undue reservation.

## Ethics Statement

The studies involving human participants were reviewed and approved by Taian Central Hospital. The patients/participants provided their written informed consent to participate in this study. The animal study was reviewed and approved by Taian Central Hospital.

## Author Contributions

HH and YaZ designed the study. SY, YiZ, YG, XL and GZ performed data collection and conducted experiments. YiZ, PL, YuZ and ZG performed data analysis and interpretation of results. YiZ, XL and SY drafted the manuscript. HH and YaZ revised the manuscript. All authors have approved the final manuscript.

## Funding

This study was supported by the Natural Science Foundation of Shandong Province, China (ZR2017MH100).

## Conflict of Interest

The authors declare that the research was conducted in the absence of any commercial or financial relationships that could be construed as a potential conflict of interest.

## Publisher’s Note

All claims expressed in this article are solely those of the authors and do not necessarily represent those of their affiliated organizations, or those of the publisher, the editors and the reviewers. Any product that may be evaluated in this article, or claim that may be made by its manufacturer, is not guaranteed or endorsed by the publisher.
