# Supplementary material for: The Association Between FGF21 and Diabetic Erectile Dysfunction: Evidence from Clinical and Animal Studies
Source: Front Endocrinol (Lausanne). 2022 Jun 15;13:874796. doi: 10.3389/fendo.2022.874796 (PMC9535403; doi:10.3389/fendo.2022.874796)
Supplement: Supplementary file 1 [file DataSheet_1.docx]

**[Supplementary material]**

**Table S1** IIEF-5 questionnaire

| **Item** | **Score** | | | | |
| --- | --- | --- | --- | --- | --- |
| 1. How do you rate your confidence that you could get and keep an erection? | Very low  1 | Low  2 | Moderate  3 | High  4 | Very high  5 |
| 1. When you had erections with sexual stimulation, how often were your erections hard enough for penetration? | Almost never/never  1 | A few times (much less than half the time)  2 | Sometimes (about half the time)  3 | Most times (much more than half the time)  4 | Almost always/always  5 |
| 1. During sexual intercourse, how often were you able to maintain your erection after you had penetrated (entered) your partner? | Almost never/never  1 | A few times (much less than half the time)  2 | Sometimes (about half the time)  3 | Most times (much more than half the time)  4 | Almost always/always  5 |
| 1. During sexual intercourse, how difficult was it to maintain your erection to completion of intercourse? | Extremely difficult  1 | Very difficult  2 | Difficult  3 | Slightly difficult  4 | Not difficult  5 |
| 1. When you attempted sexual intercourse, how often was it satisfactory for you? | Almost never/never  1 | A few times (much less than half the time)  2 | Sometimes (about half the time)  3 | Most times (much more than half the time)  4 | Almost always/always  5 |

The results over the past six months are required.

**Box 1** Calculation of sample size in human study

| We used case-control studies sample size formula to calculate the minimal required sample size based on the probability of a type I error of alpha = 5%, type II error of beta = 20% (power = 80%). In a previous study, serum FGF21 levels were normally distributed with a standard deviation (SD) of 251.73. If the true difference in the case and control means is 245.24 (case group, 482.89ng/L; control group, 237.65ng/L) (1, 2).  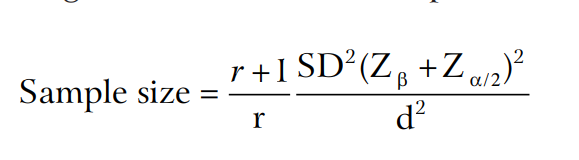  r = Ratio of control to cases, 1 for equal number of case and control  SD = Standard deviation  d = Expected mean difference between case and control  Z_β_ = Standard normal variate for power = for 80% power it is 0.84 and for 90% value is 1.28.  Z_α/2_ = Standard normal variate for level of significance as mentioned in previous section. |
| --- |


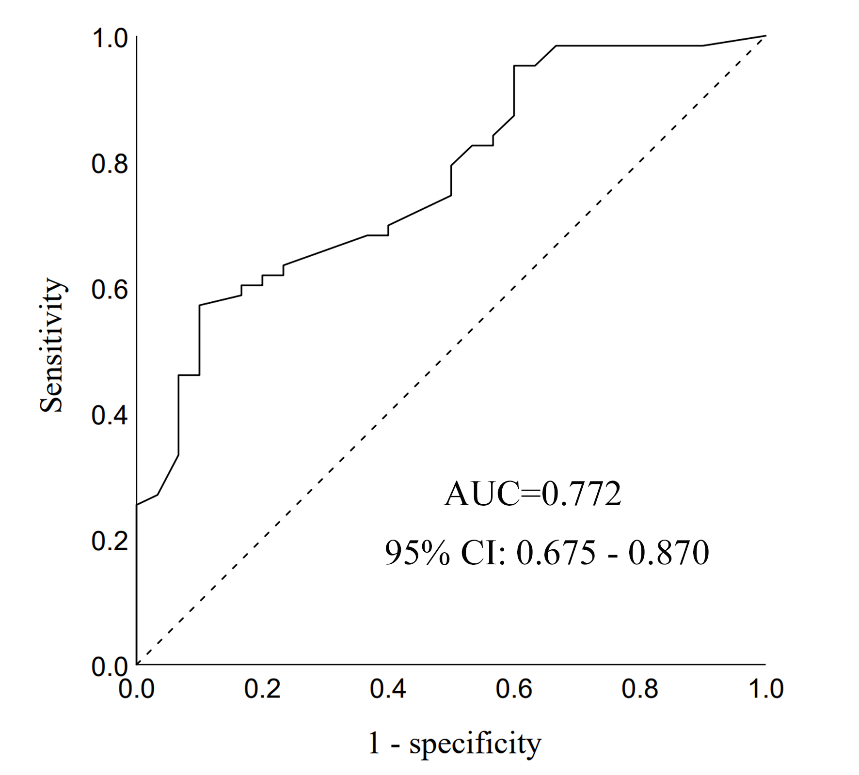


**Figure S1** Receiver operating characteristic (ROC) curve of testosterone for diagnosis of DMED. AUC, area under the curve; CI, confidence interval.

**Reference**

1. Charan J, Biswas T. How to calculate sample size for different study designs in medical research? *Indian journal of psychological medicine* (2013) 35(2):121-6. Epub 2013/09/21. doi: 10.4103/0253-7176.116232. PubMed PMID: 24049221; PubMed Central PMCID: PMCPMC3775042.

2. Lin Z, Gong Q, Wu C, Yu J, Lu T, Pan X, et al. Dynamic change of serum FGF21 levels in response to glucose challenge in human. *The Journal of clinical endocrinology and metabolism* (2012) 97(7):E1224-8. Epub 2012/04/28. doi: 10.1210/jc.2012-1132. PubMed PMID: 22539584.
